# The influence of insurance type on stage at presentation, treatment, and survival between Asian American and non‐Hispanic White lung cancer patients

**DOI:** 10.1002/cam4.1331

**Published:** 2018-03-25

**Authors:** Apichat Tantraworasin, Emanuela Taioli, Bian Liu, Raja M. Flores, Andrew J. Kaufman

**Affiliations:** ^1^ Department of Thoracic Surgery Icahn School of Medicine at Mount Sinai One Gustave L. Levy Place, Box 1023 Annenberg Building, 7‐56 New York City 10029 New York; ^2^ Department of Surgery Faculty of Medicine Chiang Mai University 110 Intawaroros Road Chiang Mai 50200 Thailand; ^3^ Department of Population Health Science and Policy and Institute for Translational Epidemiology Icahn School of Medicine at Mount Sinai One Gustave L. Levy Place, Box 1133 New York City 10029 New York; ^4^ Pharmacoepidemiology and Statistics Research Center (PESRC) Faculty of Pharmacy Chiang Mai University 239 Suthep Road Chiang Mai 50200 Thailand

**Keywords:** Disparities, medical coverage, mortality, NSCLC, SEER database

## Abstract

The effect of insurance type on lung cancer diagnosis, treatment, and survival in Asian patients living in the United States is still under debate. We have analyzed this issue using the Surveillance, Epidemiology, and End Results database. There were 102,733 lung cancer patients age 18–64 years diagnosed between 2007 and 2013. Multilevel regression analysis was performed to identify the association between insurance types, stage at diagnosis, treatment modalities, and overall mortality in Asian and non‐Hispanic White (NHW) patients. Clinical characteristics were significantly different between Asian and NHW patients, except for gender. Asian patients were more likely to present with advanced disease than NHW patients (OR_adj _= 1.12, 95% CI = 1.06–1.19). Asian patients with non‐Medicaid insurance underwent lobectomy more than NHW patients with Medicaid or uninsured; were more likely to undergo mediastinal lymph node evaluation (MLNE) (OR_adj _= 1.98, 95% CI = 1.72–2.28) and cancer‐directed surgery and/or radiation therapy (OR_adj _= 1.41, 95% CI = 1.20–1.65). Asian patients with non‐Medicaid insurance had the best overall survival. Uninsured or Medicaid‐covered Asian patients were more likely to be diagnosed with advanced disease, less likely to undergo MLNE and cancer‐directed treatments, and had shorter overall survival than their NHW counterpart.

## Introduction

According to the Surveillance, Epidemiology, and End Results (SEER) Program and the Centers for Disease Control and Prevention's National Program of Cancer Registries (CDC‐NPCR), lung cancer is the second most commonly diagnosed cancer (14% in males and 13% in female). It is also the most common cause of death in both males and females in the US (27% and 26%, respectively) 1.

Lung cancer incidence and mortality are especially high in Asian countries. In China, for example, lung cancer incidence is increasing faster than in Western countries. In 2015, the National Center Cancer Registry (NCCR) (which includes 72 local, population‐based cancer registries and covers approximately 1.37 million people) reported lung cancer incidence in China to be 733.3 per 100,000 persons and mortality to be 610.2 per 100,000 persons 2. In the United States, lung cancer incidence was 556.0 per 100,000, and mortality was 400.9 per 100,000 persons 3. In Hong Kong 4, 5, Taiwan 6, 7, 8, South Korea 9, or Japan 10, lung cancer represents the first cause of cancer death with significant increases in mortality rates over the years.

In Asian Americans, lung cancer is the most common cause of cancer death, and its incidence is second among all cancers in both genders, similar to what is observed in White, Black, Hispanic, and American Indian/Alaska natives 1. Despite the high incidence rate, Asian cancer‐specific survival rates in the United States from 2003 to 2012 were higher than in Whites and African Americans 1, 11, 12, 13.

Insurance status has been reported to be associated with lung cancer survival; however, most of these studies considered Black and White patients 14, 15, 16. To our knowledge, there are few such studies 11, 12 on Asian populations living in the United States focused specifically on patients with lung cancer. The purpose of this study is to determine the association between insurance coverage and lung cancer stage at diagnosis, cancer‐specific treatment (surgery and radiotherapy), and overall mortality in Asian patients living in the United States compared with non‐Hispanic White (NHW) patients using the SEER database.

## Materials and Methods

### Patient selection

Between 1 January 2004 and 31 December 2013, there were 447,167 patients diagnosed with lung cancer and identified in the public‐use SEER database (SEER*Stat software version 8.3.2, Calverton, MD) 17. The information on insurance type was not recorded until 2007; therefore, the database was restricted to 2007 onward (*n* = 318,951). Patients who were aged <18 years (*n* = 28 patients) or >64 years (*n* = 216,190) at diagnosis were excluded because the designation of insurance type in the database is unreliable for patients ≥65 years of age. The number of patients included in this data analysis was 80,885 (74,301 for NHW and 6584 for Asian patients).

The Icahn Medical School at Mount Sinai Review Board for Health Sciences Research considered this study “exempt.”

### Variables selection

Patient characteristics included age, race, gender, marital status, insurance type, rural–urban residence, percent of county below poverty, and histologic or pathologic reports. The definition of Asian included Asian or Pacific Islander. Marital status was categorized in three levels: “single,” “unmarried/domestic partner or married,” and “divorced/separated or widow.” Rural included less urban or rural areas and urban included big metropolitan, metropolitan, or urban areas.

Insurance type was defined as: uninsured (not insured, self pay); Medicaid (any Medicaid including Indian/Public Health Service, Medicaid, Medicaid‐administered through a managed care plan, or Medicare with Medicaid eligibility); and non‐Medicaid (insured including private insurance, Fee‐for‐Service, Managed care, HMO‐Health maintenance organizations, PPO‐Preferred provider organizations, TRICARE, insured/no specifics, Medicare‐administered through a managed care plan, Medicare with private supplement, Medicare with supplement, NOS and Military, or no specifics Medicare/Medicare‐NOS). Patients with unknown insurance type were excluded from the multivariable analysis.

Percent of county below federal poverty was obtained from linked county‐level data 18. The ICD‐0‐3 SEER site/histology validation list was used for extracting the information of tumor histology which was then categorized into two groups: small cell carcinoma and non‐small cell carcinoma (NSCLC). Non‐small cell lung cancer was subcategorized into three groups: Adenocarcinoma, squamous cell carcinoma, and others. Tumor staging was based on the 6th edition of the American Joint Committee on Cancer Staging Atlas. Extent of disease was based on TNM staging and categorized as localized (Stage I, no nodal or metastatic disease), regional (stage II or III, nodal disease), or distant (stage IV, any metastatic disease), as previously described by Walker et al. 19.

### Treatments and outcomes

Radiation therapy (RT) and surgery were identified based on SEER variables. Radiotherapy was defined as beam radiation (SEER codes: beam radiation, combination of beam with implants or isotopes, radioactive implants, or radioisotopes). Cancer‐directed surgery was defined using SEER surgical codes: surgery at primary site— partial, wedge, segmentectomy, partial lobectomy, sleeve resection, lobectomy, bilobectomy, complete/total/standard/extended/radical pneumonectomy, and recommended for surgery. Cancer‐directed surgery and/or radiation therapy were defined as surgery and/or radiation therapy related to primary lung cancer. Mediastinal lymph node evaluation (MLNE) was defined as regional lymph node removed for examination with or without presurgical systemic treatment or radiation.

The primary outcome was overall mortality. Cancer‐specific death or cause‐specific death on death certificates was not utilized because they may be subject to misclassification when compared to other approaches 20, 21. Moreover, cause‐specific death may lead to underestimation of mortality resulting from the disease of interest in the presence of competing causes of death. Survival time was defined as the time between diagnosis and death or date of last follow‐up, through 31 December 2013.

### Statistical analysis

For univariable analyses, Pearson's chi‐square test was used to assess the significance of the difference between proportions. Student's *t*‐test or rank sum test was used to assess the significance of the difference between means. The associations between insurance type and overall mortality were studied using adjusted parametric survival curves analyzed by mixed‐effects Weibull regression.

A multilevel logistic regression model (adjusted for age, marital status, rural–urban area, percent of county below poverty, pathologic results, and stage of disease, and analyzed under stratification of identical SEER registry where patients lived) was used to identify the association between insurance type and surgical procedure (lobectomy vs. sublobar resection) according to race in stage I‐III patients. The analysis on the association between insurance status and cancer‐directed surgery and/or radiotherapy (RT) excluded patients diagnosed with stage IV NSCLC because the primary treatment for these patients is usually chemotherapy and these data were not available in SEER. The adjusted odds ratio (OR_adj_) with 95% confidence interval (CI) is reported.

The multilevel parametric survival model analyzed under stratification of identical SEER registry was used with multivariable analyses to determine the effect of insurance type on overall mortality. The estimated hazard ratio (HR) with 95% CI is reported. Multiple imputations with multivariate normal equation were performed for any variable that had at least 10% missing values. The results were compared to those from a complete‐case analysis, and if they were similar, the results from the complete‐case analysis were reported 22.

All tests were two‐sided. A *P*‐value <0.05 was considered statistically significant. STATA program version 14.0 (StataCorp, College Station, TX) was used for statistical analysis.

## Results

There were 80,885 patients in this study: 6584 Asian and 74,301 NHW. Patient characteristics differ between groups except for gender. Asian patients were younger at diagnosis, more likely to be married, live in rural areas, in high‐income counties, diagnosed with adenocarcinomas, have stage IV cancer, have a lower lobe tumor location, a more differentiated tumor, undergo mediastinal lymph node evaluation, and were treated with lobectomy in comparison with NHW patients. Asian patients were less likely to be uninsured, divorced, or separated, and be treated with cancer‐directed surgery and/or radiation therapy in stage I‐III (Table 1).

**Table 1:** Patient characteristics (SEER 2007–2013)

Variable	*N* (%)	Asian *N* (%)	NHW *N* (%)	*P*‐value
Number of patients	80,885	6584	74,301	
Age, years (mean ± SD)	56.7 ± 6.2	55.7 ± 7.2	57.0 ± 6.0	<0.001
Gender				
Female	37,695 (46.6)	3009 (45.7)	34,686 (46.7)	0.128
Male	43,190 (53.4)	3575 (54.3)	39,615 (53.3)	
Insurance type				
Non‐Medicaid	58,685 (72.6)	4664 (70.8)	54,021 (72.7)	<0.001
Medicaid	14,376 (17.8)	1352 (20.5)	13,024 (17.5)	
Uninsured	5936 (7.3)	400 (6.1)	5536 (7.5)	
Unknown	1888 (2.3)	168 (2.6)	1720 (2.3)	
Marital status				
Single	14,039 (17.4)	996 (15.1)	13,043 (17.5)	<0.001
Unmarried/domestic partner or married	44,241 (54.7)	4389 (66.7)	39,852 (53.6)	
Divorced/separated/widow	19,307 (23.9)	920 (14.0)	18,387 (24.8)	
Unknown	3298 (4.0)	279 (4.2)	3019 (4.1)	
Rural–urban area				
Urban	12,437 (15.4)	273 (4.2)	12,164 (16.4)	<0.001
Rural	68,282 (84.4)	6149 (93.4)	62,133 (83.6)	
Unknown	166 (0.2)	162 (2.4)	4 (0.01)	
Percent of county below poverty				
<10	11,807 (14.6)	1662 (25.3)	10,145 (13.7)	<0.001
10–12.99	20,526 (25.4)	1818 (27.6)	18,708 (25.2)	
13–16.49	12,490 (15.4)	883 (13.4)	11,607 (15.6)	
≥16.5	36,058 (44.6)	2221 (33.7)	33,837 (45.5)	
Histology				
Small cell carcinoma	12,723 (15.7)	473 (7.2)	12,250 (16.5)	<0.001
Non‐small cell carcinoma (NSCLC)				
Adenocarcinoma	34,915 (43.2)	3998 (60.7)	30,917 (41.6)	
Squamous cell carcinoma	14,812 (18.3)	764 (11.6)	14,048 (18.9)	
Others	18,435 (22.8)	1349 (20.5)	17,086 (23.0)	
Location of tumor				
Upper lobe	42,530 (52.6)	3248 (49.3)	39,282 (52.9)	<0.001
Middle lobe	3395 (4.2)	350 (5.3)	3045 (4.1)	
Lower lobe	18,208 (22.5)	1693 (25.7)	16,515 (22.2)	
Main bronchus	4749 (5.9)	253 (3.8)	4496 (6.1)	
Overlap lesion	1062 (1.3)	85 (1.3)	977 (1.3)	
Non‐specified	10,941 (13.5)	955 (14.6)	9986 (13.4)	
Tumor differentiation				
Well differentiated	3637 (4.5)	434 (6.6)	3203 (4.3)	<0.001
Moderately differentiated	12,329 (15.2)	1145 (17.4)	11,184 (15.1)	
Poorly differentiated	20,937 (25.9)	1623 (24.7)	19,314 (26.0)	
Undifferentiated	3494 (4.3)	154 (2.3)	3340 (4.5)	
Unknown	40,488 (50.1)	3228 (49.0)	37,260 (50.1)	
Laterality				
Right	45,088 (55.7)	3683 (56.0))	41,405 (55.7)	0.011
Left	31,590 (39.1)	2548 (38.7)	29,042 (39.1)	
Bilateral	3833 (4.7)	338 (5.1)	3495 (4.7)	
Unknown unilateral	374 (0.5)	15 (0.2)	359 (0.5)	
Stage of disease				
IA	8555 (10.6)	669 (10.2)	7886 (10.6)	<0.001
IB	6123 (7.6)	452 (6.9)	5671 (7.6)	
IIA	898 (1.1)	69 (1.1)	829 (1.1)	
IIB	2733 (3.4)	183 (2.8)	2550 (3.4)	
IIIA	7766 (9.6)	523 (7.9)	7243 (9.8)	
IIIB	12,230 (15.1)	977 (14.8)	11,253 (15.2)	
IV	42,580 (52.6)	3711 (56.3)	38,869 (52.3)	
Cancer‐directed surgery				
No surgery	2481 (11.0)	151 (8.3)	2330 (11.2)	<0.001
Sublobar resection	3906 (17.3)	256 (14.0)	3650 (17.6)	
Lobar resection	14,474(64.0)	1314 (72.0)	13,160 (63.4)	
Pneumonectomy	1531 (6.8)	90 (4.9)	1441 (6.9)	
Unknown surgical type	202 (0.9)	13 (0.7)	189 (0.9)	
Mediastinal lymph node evaluation				
No	59,521(73.6)	4787 (72.7)	54,734 (73.7)	0.041
Yes	18,631 (23.0)	1542 (23.4)	17,089 (23.0)	
Unknown	2733 (3.4)	255 (3.9)	2478 (3.3)	
Cancer‐directed surgery and/or radiation therapy^a^				
No	7135 (18.6)	628 (21.9)	6507 (18.4)	<0.001
Yes	31,097 (81.2)	2244 (78.0)	28,853 (81.4)	
Unknown	73 (0.2)	1 (0.1)	72 (0.2)	
Overall mortality				
Survived	25,999 (32.1)	2706 (41.1)	23,293 (31.3)	<0.001
Death	54,886 (67.9)	3878 (58.9)	51,008 (68.7)	
Follow time (months), Median (IQR)	9 (3–23)	11 (4–27)	9 (3–22)	<0.001

NHW, non‐Hispanic White.

a Stage IV patients were excluded from subsequent analyses.

### Stage at diagnosis

**Figure 1:**
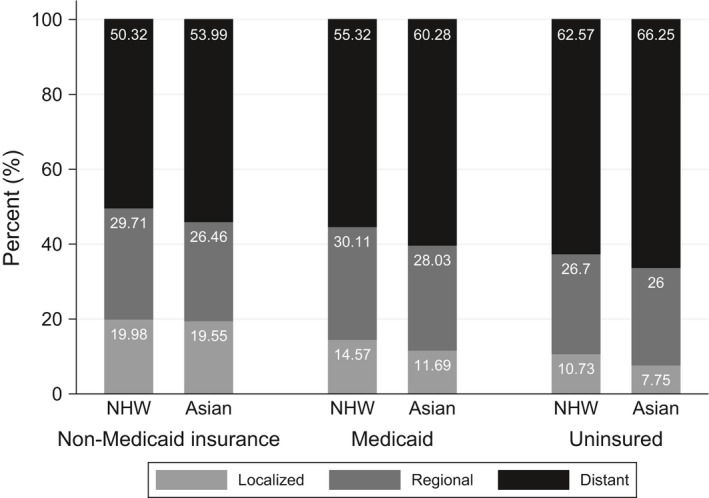
Proportion of patients presenting with localized, regional, or distant disease at time of diagnosis by race and insurance status. All *P* < 0.001. Stage I disease was considered localized, stage II to III disease was considered regional, and stage IV disease was considered distant.

Uninsured patients were more likely to present with distant disease than those with Medicaid and non‐Medicaid insurance across all race groups. Patients with non‐Medicaid insurance were more likely to present with localized disease than those uninsured or with Medicaid coverage (Fig. 1). Asian patients were more likely to present with advanced disease than NHW patients (OR_adj _= 1.12, 95% CI = 1.06–1.19). The proportion of patients presenting with localized, regional, or distant disease according to race and insurance status in each poverty group was similar (Appendix A). However, Asian patients living in a high‐income county (percent of county below poverty <10%) were less likely to present with advanced disease than NHW patients (OR_adj _= 0.48, 95% CI = 0.42–0.55) (Appendix B).

After adjusting for age, gender, marital status, rural–urban area, and percent of county below poverty, the odds (OR_adj_) of advanced stage at the time of diagnosis for uninsured and Medicaid patients compared to non‐Medicaid patients were 1.64 (95% CI = 1.32–2.05 and 1.31 (95% CI = 1.15–1.49) for Asian patients and 1.63 (1.53–1.73) and 1.21 (1.16–1.26) for NHW patients. There was no significant interaction between race and insurance type (*P*‐value = 0.205). The analysis according to poverty group shows that uninsured patients living in high‐income counties were less likely to present with advanced diseased than non‐Medicaid patients (OR_adj _= 0.80, 95% CI = 0.67–0.96) (Appendix B).

### Cancer‐directed treatment

#### Type of cancer‐directed surgery

The analysis of stage I‐III patients shows that the percentage of patients undergoing lobectomy among those with non‐Medicaid insurance, uninsured, and Medicaid were 82.5%, 83.2%, and 78.6%, respectively (*P* < 0.001) (not shown in the table). In the multivariable analysis, patients with Medicaid coverage were less likely to undergo lobectomy compared to uninsured patients (Table 2). These results were similar when stratified by poverty status, but the differences were not statistically significant (Appendix C). Asians were more likely to undergo a lobectomy, independently from insurance and poverty status.

**Table 2:** Odds of lobectomy, mediastinal lymph node (MLN) evaluation, and cancer‐specific surgery and/or radiotherapy according to race and insurance type^a^

Variables	Lobectomy		MLN evaluation		Cancer‐directed surgery/RT	
	OR_adj_	95% CI	OR_adj_	95% CI	OR_adj_	95% CI
Race						
Non‐Hispanic White (NHW)	1.00	Reference	1.00	Reference	1.00	Reference
Asian	1.50	1.26–1.80	0.98	0.89–1.07	0.80	0.72–0.89
Insurance type						
Uninsured	1.00	Reference	1.00	Reference	1.00	Reference
Non‐Medicaid	0.94	0.76–1.17	1.96	1.77–2.17	1.73	1.56–1.92
Medicaid	0.72	0.57–0.91	1.11	0.99–1.24	1.08	0.97–1.21
Race & Insurance type						
NHW & Uninsured	1.00	Reference	1.00	Reference	1.00	Reference
NHW & Non‐Medicaid	0.97	0.78–1.20	1.90	1.72–2.12	1.66	1.49–1.86
NHW & Medicaid	0.76	0.59–0.96	1.11	0.98–1.24	1.05	0.93–1.18
Asian & Uninsured	2.89	0.86–9.57	0.70	0.47–1.05	0.50	0.35–0.72
Asian & Non‐Medicaid	1.50	1.12–2.00	1.98	1.72–2.28	1.41	1.20–1.65
Asian & Medicaid	0.88	0.57–1.38	0.90	0.72–1.12	0.83	0.66–1.03

a Stage IV patients were excluded from data analysis, and multivariable model was adjusted for age, marital status, rural–urban area, percent of county below poverty, pathology results, and stage of disease, and analyzed under stratification of identical state by multilevel logistic regression model.

There was no statistically significant difference in surgical procedures in each stage of disease between uninsured or Medicaid‐covered Asians and NHWs; however, in patients with non‐Medicaid coverage, lobectomy was more frequent in Asian patients (Table 3). There was no significant interaction between race and insurance types for this outcome (*P*‐value = 0.375).

**Table 3:** Type of cancer‐directed surgery according to race and insurance type

	Uninsured			Medicaid			Non‐Medicaid		
	Asian	NHW	*P*‐value	Asian	NHW	*P*‐value	Asian	NHW	*P*‐value
Stage I									
Sublobar resection	2 (8.7)	66 (15.7)	0.651	20 (17.7)	264 (22.2)	0.496	102 (12.6)	1644 (18.8)	<0.001
Lobectomy	20 (87.0)	339 (80.5)		90 (79.7)	881 (74.3)		692 (85.8)	6827 (78.1)	
Pneumonectomy	1 (4.4)	16 (3.8)		3 (2.6)	42(3.5)		13 (1.6)	275 (3.1)	
Stage II									
Sublobar resection	0	7 (6.7)	0.282	2 (6.9)	26 (8.4)	0.383	7 (4.5)	143 (7.4)	0.001
Lobectomy	7 (100)	71 (67.6)		25 (86.2)	230 (74.7)		140 (90.3)	1469 (76.2)	
Pneumonectomy	0	27 (25.7)		2 (6.9)	52 (16.9)		8 (5.2)	317 (16.4)	
Stage III									
Sublobar resection	1 (6.3)	34 (21.0)	0.229	10 (19.6)	98 (21.6)	0.685	24 (8.5)	507 (16.5)	0.001
Lobectomy	13 (81.2)	94 (58.0)		35 (68.6)	283 (62.3)		216 (76.3)	2088 (68.1)	
Pneumonectomy	2 (12.5)	34 (21.0)		6 (11.8)	73 (16.1)		43 (15.2)	473 (15.4)	

NHW, non‐Hispanic White.

#### Cancer‐directed surgery and/or radiotherapy

Uninsured patients were less likely to undergo cancer‐directed surgery and/or radiotherapy (RT). The percentage undergoing cancer‐directed surgery and/or RT in uninsured, non‐Medicaid, and Medicaid coverage was 73.4%, 83.6%, and 74.1%, respectively (*P* < 0.001) (not shown in the table). In the multivariable analysis, patients with non‐Medicaid insurance were more likely to undergo cancer‐directed surgery and/or RT compared to uninsured patients (Table 2). These results were similar in each poverty group (Appendix C). Asian patients were less likely to be treated with cancer‐directed surgery and/or RT than NHW patients. However, the statistically significant difference was shown only in patients living in poor‐income county (percent of county below poverty ≥16.5) (Appendix C).

There was no significant interaction between race and insurance type (*P*‐value = 0.368). Those with non‐Medicaid insurance were more likely to receive cancer‐directed surgery and/or RT independently from race. These results were similar in each poverty group (Appendix C).

#### Mediastinal lymph node evaluation (MLNE)

Patients with non‐Medicaid insurance or Medicaid coverage most often underwent MLNE. Asian patients were more likely to undergo MLNE than NHW patients at the univariable analysis; however, the differences were not statistically significant in the multivariable model. There was no significant interaction between race and insurance type for this outcome (*P*‐value = 0.119). Those with non‐Medicaid insurance were more likely to undergo MLNE independently from race. These results were similar in each poverty group (Appendix C).

### Overall mortality

Patients with Medicaid or uninsured status had a higher mortality compared to those with non‐Medicaid insurance. Overall mortality was significantly lower in Asian patients compared to NHW patients in a multilevel parametric survival model adjusted for patient characteristics, insurance status, stage of disease, and treatments (Table 4).

**Table 4:** Hazard ratios of overall mortality for ethnicity, insurance type and interaction between ethnicity and insurance type, analyzed by multilevel parametric survival analysis adjusted by patient demographic, pathologic result, stage of disease, and cancer‐specific treatment

Overall mortality	HR_adj_	95% CI
Race		
Non‐Hispanic White	1.00	Reference
Non‐Hispanic Black	1.00	0.96–1.03
American Indian/Alaska	1.05	0.86–1.27
Hispanic	0.85	0.80–0.89
Asian	0.66	0.63–0.70
Insurance type		
Non‐Medicaid insurance	1.00	Reference
Medicaid	1.34	1.29–1.38
Uninsured	1.24	1.18–1.30
Race and Insurance type		
Non‐Hispanic White with uninsured	1.00	Reference
Non‐Hispanic White with non‐Medicaid	0.76	0.72–0.81
Non‐Hispanic White with Medicaid	1.05	0.98–1.12
Non‐Hispanic Black with uninsured	0.93	0.83–1.03
Non‐Hispanic Black with non‐Medicaid	0.78	0.72–0.83
Non‐Hispanic Black with Medicaid	1.01	0.93–1.10
American Indian/Alaska with uninsured	1.62	0.73–3.63
American Indian/Alaska with non‐Medicaid	0.72	0.55–0.93
American Indian/Alaska with Medicaid	1.23	0.90–1.69
Hispanic with uninsured	0.72	0.60–0.85
Hispanic with non‐Medicaid	0.67	0.62–0.74
Hispanic with Medicaid	0.86	0.77–0.96
Asian with uninsured	0.54	0.44–0.68
Asian with non‐Medicaid	0.52	0.47–0.56
Asian with Medicaid	0.67	0.59–0.76

**Figure 2:**
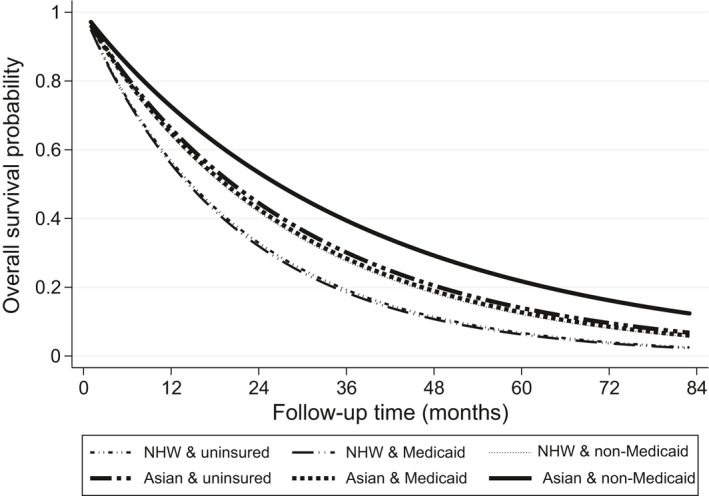
Adjusted parametric survival curves illustrating cancer‐specific survival by insurance status for Asian and non‐Hispanic White lung cancer patients living in the United States analyzed by mixed‐effects Weibull regression. *P* < 0.05.

There were significant interactions between race and insurance type for overall mortality (*P*‐value <0.001). Asian patients with non‐Medicaid coverage had the best overall survival (Fig. 2, *P* < 0.001). The hazard ratios of overall mortality for interaction between race and insurance type (Table 4) show that uninsured patients or Medicaid patients had worse survival compared to those with non‐Medicaid coverage independently from race. However, Asian with any insurance status had better overall survival than NHW with any insurance status. These associations among race, insurance status, and overall survival were similar across groups of poverty (Appendices D and E). Among non‐Asian patients, Hispanic patients with non‐Medicaid insurance had the best overall survival compared with uninsured NHW. There was no difference in overall mortality between non‐Hispanic Black and NHW. However, non‐Hispanic Black with non‐Medicaid insurance had better overall survival than uninsured NHW.

Other comparative prognostic factors for overall mortality in patients with Asian lung cancer included older age, male gender, higher percent of county below poverty, poorer tumor differentiation, and higher stage of disease. The favorable prognostic factors for overall survival were adenocarcinoma cell type rather than squamous cell carcinoma, being married or with a domestic partner, and receiving MLNE with cancer‐directed surgery and/or RT. Among NHW patients, prognostic factors for overall mortality were the same as Asian patients. In addition, patients living in rural areas had better overall survival compared to those living in urban areas (Table 5).

**Table 5:** Prognostic factors for overall mortality in Asian and non‐Hispanic White non‐small cell lung cancer living in the United States analyzed under stratification of identical state by multilevel parametric survival model

Variables	Asian		Non‐Hispanic White	
	Hazard Ratio	95% CI	Hazard Ratio	95% CI
Insurance				
Non‐Medicaid	1.00	Reference	1.00	Reference
Medicaid	1.31	1.21–1.42	1.30	1.27–1.33
Uninsured	1.28	1.10–1.49	1.27	1.23–1.32
Age	1.01	1.01–1.02	1.01	1.01–1.02
Male	1.36	1.26–1.46	1.26	1.23–1.28
Percent of county below poverty (continuous variable)	1.01	1.01–1.02	1.01	1.01–1.02
Marital status				
Single	1.00	Reference	1.00	Reference
Married or domestic partner	0.89	0.81–0.98	0.89	0.87–0.91
Divorced/separate/widow	0.95	0.84–1.08	1.04	1.01–1.07
Rural‐urban area				
Urban	1.00	Reference	1.00	Reference
Rural	1.13	0.98–1.32	0.97	0.94–0.99
Histology				
Squamous cell carcinoma	1.00	Reference	1.00	Reference
Adenocarcinoma	0.65	0.58–0.72	0.84	0.82–0.86
Small cell carcinoma	0.86	0.73–1.01	0.89	0.86–0.91
Others	0.90	0.79–1.01	0.95	0.93–0.98
Location of tumor				
Upper lobe	1.00	Reference	1.00	Reference
Middle lobe	0.96	0.81–1.13	0.95	0.91–1.01
Lower lobe	0.96	0.88–1.04	1.00	0.98–1.03
Main bronchus	1.22	1.04–1.44	1.14	1.10–1.18
Overlap lesion	1.07	0.80–1.44	1.05	0.97–1.14
Tumor differentiation				
Well differentiated	1.00	Reference	1.00	Reference
Moderately differentiated	1.41	1.12–1.77	1.38	1.28–1.48
Poorly differentiated	1.85	1.49–2.31	1.76	1.64–1.88
Undifferentiated	2.09	1.56–2.79	1.75	1.62–1.90
Laterality				
Right	1.00	Reference	1.00	Reference
Left	1.02	0.95–1.10	1.01	0.99–1.03
Bilateral	0.97	0.82–1.15	0.98	0.93–1.03
Stage of disease (NSCLC)				
IA	1.00	Reference	1.00	Reference
IB	1.86	1.38–2.51	1.46	1.37–1.56
IIA	2.13	1.28–3.54	1.67	1.48–1.89
IIB	3.59	2.59–4.98	2.09	1.94–2.25
IIIA	3.56	2.73–4.64	2.50	2.36–2.65
IIIB	4.22	3.28–5.45	3.09	2.93–3.26
IV	7.58	5.93–9.70	5.87	5.58–6.18
Mediastinal lymph node evaluation	0.47	0.41–0.53	0.49	0.47–0.51
Cancer‐directed surgery and/or radiation therapy^a^	0.65	0.55–0.76	0.53	0.51–0.56

a Stage IV was excluded in multilevel parametric survival model.

Type of cancer‐directed surgery variable was not included in multivariable analysis model because of multicollinearity with cancer‐directed surgery and/or radiation therapy.

Subgroup analysis of patients diagnosed with adenocarcinoma analyzed by multilevel parametric survival model, adjusted by patient demographic, stage of disease, and cancer‐directed treatment, showed the same effects of insurance type and race on overall survival (Appendix F).

## Discussion

This study analyzed the relationship between insurance type and stage of disease at diagnosis, treatment, and overall mortality among adult Asian and NHW patients diagnosed with lung cancer using the SEER data set.

Firstly, we found that Asian patients had different characteristics compared with NHW patients: younger, less uninsured status, higher married status, more living in rural areas, less percent of county below poverty, more advanced stage at diagnosis, and less underwent MLNE and cancer‐directed surgery and/or RT. Secondly, we found that race and insurance status affected lung cancer stage at diagnosis, types of surgery, MLNE, cancer‐directed surgery and/or RT, and overall mortality as described above.

There are some disparities in several aspects of lung cancer patients including stage at presentation, treatment selection, and overall survival or cancer‐specific mortality which have been associated with patient race and insurance status 19, 23, 24, 25, 26, 27, 28. However, these studies report the disparities comparing African American and White patients. Data from the American Community Survey (ACS) and the United States Census Bureau, Census Information Centers (CIC) demonstrated that the Asian population is 5.6% of the total population in the United States in 2003. The Asian population increased 47% from 2005 (13,879,891) to 2015 (20,416,808) 29. Furthermore, data from the SEER database, the Centers for Disease Control and Prevention (National Program of Cancer Registries, NPCR), and the North American Association of Central Cancer Registries reported that lung cancer has the second highest incidence rate (male 47.4 per 100,000 population, female 28.3 per 100,000 population) and the highest mortality rate (male 34.0 per 100,000 population, female 18.2 per 100,000 population) in Asian/Pacific Islander patients in both male and female (data from 2008 to 2012) 1. Therefore, lung cancer is very important not only in White and Black populations but also in Asian populations living in the United States. The data from The National Cancer Data Base 27 and the Florida Cancer Data System Registry to the Florida's Agency for Health Care Administration and the US Census 30 found that Asian lung cancer patients had longer survival than White patients after pulmonary resection. Their findings are similar to our present study. The current study showed that the mortality rate of Asian lung cancer patients was lower than that of White and Black patients 1, 27. Therefore, the risk factors which impact this racial disparity should be further explored.

Several previous studies demonstrated that insurance status had an effect on survival in lung cancer patients. Among patients <65 years, previous studies found that Medicaid or uninsured status was associated with higher mortality compared with non‐Medicaid status 14, 31, 32. The cause of the disparity in lung cancer care or outcomes based on insurance status are likely multifactorial including patient factors such as race, poverty, incomes, and healthcare system. Three possible reasons correlated with insurance status that may impact lung cancer survival: (1) Accessibility of patient care; several previous studies using statewide administrative databases demonstrated that patients with non‐Medicaid or private insurance were more likely to be treated at high‐volume hospitals compared to patients with Medicaid and uninsured status 33, 34. (2) Stage of disease at presentation or diagnosis; patients with non‐Medicaid status were less likely to present with distant disease and more likely to present with localized disease compared with any insurance status 19. Our study had the same result. However, in the analysis according to poverty group, Asian patients living in high‐income counties (percent county below poverty <10%) were less likely to present with advanced disease at time of diagnosis. This result may be explained by the highest proportion of non‐Medicaid status in this group (Appendix G). (3) Surgical procedures or other treatment modalities; patients uninsured or with Medicaid were significantly less likely than those with non‐Medicaid to undergo a lobectomy for early‐stage NSCLC 15. The result from this study also confirmed that Asian patients with non‐Medicaid were more likely to undergo lobectomy than those uninsured or on Medicaid. Walker et al. reported that among non‐metastatic patients, patients with non‐Medicaid status were more likely to undergo cancer‐directed surgery and/or receive RT compared with those without insurance coverage 19. Our study demonstrated the same results, both in Asian and NHW patients. Esnaola et al. compared surgical resection for localized NSCLC among Whites and African Americans in South Carolina, and reported that patients with uninsured, Medicare or Medicaid status were less likely to undergo pulmonary resection compared with commercial insurance 35. It is known that LN dissection/or sampling (MLNE) has become the standard of care during curative lung resection in NSCLC 36, 37, 38, 39. We found that Asian and NHW patients with non‐Medicaid insurance were more likely to undergo MLNE than those with uninsured status. Therefore, insurance status was a significant prognostic factor for lung cancer stage at diagnosis, treatment, and survival. However, one of the caveats of the insurance records that we used for this study is that we could not accurately distinguish patients who were covered by Medicaid at the time of diagnosis and those who were uninsured at diagnosis but enrolled in Medicaid at that time—this is common practice by hospitals when individuals qualify to ensure that they recover some of the healthcare costs. In both of these scenarios, the patient would be listed in the database as having Medicaid coverage.

The data from the ACS demonstrated that the Asian population in the United States gradually increased from 13 million in 2005 to 20 million in 2015, approximately 5.6% of the total population of the United States. In 2015, the proportion of the Asian population who had uninsured status, Medicaid, and non‐Medicaid was 7.5%, 25.0%, and 73.6%, respectively 29. The number of people with insurance is increasing while non‐insurance is decreasing. This proportion of insurance status is concordant with those of Asian lung cancer patients. In this study, the proportion of Asian lung cancer patients with uninsured, non‐Medicaid, and Medicaid status is 6.1%, 70.8%, and 20.5%, respectively, and that of NHW patients is 7.5%, 72.7%, and 17.5% respectively. The proportion of Asian patients having any insurance coverage is significantly higher than those of NHW patients. This finding may explain why the survival of Asian patients is longer than that of NHW patients. Ou et al. reported a retrospective population‐based study of NSCLC cases from the cancer surveillance programs of three Southern California counties from 1991 to 2005 and found that Asian ethnicity was an independent favorable prognostic factor for overall survival in NSCLC regardless of smoking status (HR = 0.86, 95% CI = 0.81–0.92) 40.

Although most of the independent prognostic factors for overall mortality are the same between Asian and NHW patients, we found some interesting points. Asian patients had significantly higher tumor differentiation, higher stage at presentation, and less cancer‐directed surgery and/or RT; but they had better overall survival. Although some of the late stage at presentation and disease treatment patterns may be explained by the proportion of insured patients as described above, other independent prognostic factors should be considered such as genetic or immune disparities among race of patients. Alternative medical practice or any cultural component may affect lung cancer treatment especially in Asian patients 41. An unproved or disproved alternative medicine approach may correlate with higher stage at presentation and delay or impaired treatment 42. This information is not available in the SEER database.

To our knowledge, the relationship among insurance status and stage at diagnosis, treatment, and overall mortality in Asian NSCLC patients living in the United States has never been deeply explored. We also found that insurance status does influence stage at the time of lung cancer diagnosis, treatment, and outcome as discussed above. Non‐Medicaid patients were more likely to undergo cancer‐directed surgery and/or receive RT, and less likely to die compared with uninsured patients, while Medicaid patients had intermediate outcome.

The strength of this study is that this is we reported specifically on Asian American lung cancer patients which identifies the effect of insurance status on lung cancer diagnosis, treatment, and outcomes using overall mortality as a primary outcome. This is different from previous studies that also used the SEER database 11, 12, 13, 19. As we mentioned in the Method section, cancer‐specific mortality and cause of death from the death certificates relied on the judgment and interpretation of physicians who evaluated cause of death; sometimes it is difficult to distinguish between cancer‐specific and cancer‐consequent deaths 43. Many previous studies described the potential biases introduced by misclassification of cause‐of‐death and disadvantages in using “cause‐specific death” as a primary outcome for population‐based survival approach 20, 21, 43, 44, 45. We used multilevel parametric survival analysis which simultaneously examines the effects of individual‐level and group‐level factors to assess the effect of insurance status on overall survival adjusted for confounding factors and the SEER registry (as a surrogate of state) where patients lived. We assumed that each SEER registry represents a state that has different characteristics of health care and personnel. Healthcare variables such as hospital volume, hospital type, or surgeon volume are not available in the SEER database, thus we assumed that patients living in the same state had access to a similar healthcare system. Because each individual subject is nested in a group level of SEER registry, a multilevel analysis is more appropriate than a traditional regression which assumes independence of each individual level. This method analysis is statistically more efficient than the traditional Cox's proportion hazard model, especially in a large population‐based database.

The limitation of this study involves a function of the data set used. The SEER registry does not include some previously reported variables that may influence the diagnosis, treatment, and survival of lung cancer patients such as hospital types 32, smoking status 46, performance status 46, medical comorbidities 47, surgeon volume 48, hospital volume 48, 49, 50, 51, complementary and alternative medicine practices 42, and use of systemic chemotherapy, which could not be adjusted in the data analysis. Moreover, the SEER insurance variable does not subdivide those in Medicare and coverage from the military or Veterans Affairs 19. There is no information about time of enrollment in Medicaid coverage or other insurance that might affect stage of disease at presentation and survival. Bradley et al. 52 and Koroukina et al. 53 reported that patients enrolled in Medicaid around the time of diagnosis presented with more advanced disease and had worse survival compared to those who were previously enrolled in Medicaid. Finally, because of non‐specificity of Medicare status in patients ≥65 years in the SEER data set, only 32.7% of patients in SEER database were included in this study; therefore, we could not make conclusions referred to patients age ≥65 years.

The results of this study found that non‐Medicaid insurance affected cancer care and mortality. Consequently, the next question for exploration should be what variables create these differences when compared to Medicaid and uninsured status. The barriers to insurance for uninsured patients should also be explored. Currently, lung cancer screening has been accepted in high‐risk patients. This will be important for early diagnosis, treatment, and survival. Whenever most insurance types include lung cancer screening with low‐dose CT scan in health programs, the effect of insurance type to lung cancer diagnosis, treatment, and outcome is more apparent. To improve patient survival, insurance coverage should be promoted for those with uninsured status.

## Conclusions

In lung cancer patients living in the United States, lack of insurance is associated with advanced disease at presentation and less cancer‐directed surgery and/or RT compared to non‐Medicaid insurance independently from race (Asian or NHW). Patients with non‐Medicaid insurance were more likely to be diagnosed at an early stage at presentation, receive cancer‐directed treatment, and lymph node evaluation, and have better overall survival. Upcoming policy changes resulting in low‐dose CT screening as a requirement in non‐Medicaid and Medicaid insurance will alter the stage of lung cancer at diagnosis, treatment, and overall survival in the United States. There are some correlations between race (Asian and NHW) and insurance status, and an effect on overall mortality. Asian patients with or without any insurance had a better survival than uninsured NHW, but only NHW with non‐Medicaid insurance had a longer survival than uninsured NHW. The benefit of Medicaid insurance in the United State should be explored further. More studies should be performed to identify the other factors that may relate to health disparities in lung cancer patients such as cost of treatment covered, drug and tests covered, and copayment.

## Conflict of Interest

All authors have no relevant conflict of interests.

## Appendix A

Proportion of patients presenting with localized, regional, or distant diseases according to race and insurance status in each poverty group. All *P* < 0.001.

## Appendix B

**Table:** Odds of presenting with advanced disease at the time of diagnosis according to race and insurance type in each poverty group

Percent of county below poverty	<10	10–12.99	13.00–16.49	≥16.5
	OR_adj_ (95%CI)	OR_adj_ (95%CI)	OR_adj_ (95%CI)	OR_adj_ (95%CI)
Race				
Non‐Hispanic White	Reference	Reference	Reference	Reference
Asian	0.48 (0.42–0.55)	1.17 (1.05–1.31)	1.14 (0.98–1.32)	1.12 (1.02–1.23)
Insurance type				
Non‐Medicaid	Reference	Reference	Reference	Reference
Medicaid	1.16 (0.96–1.36)	1.34 (1.23–1.46)	1.16 (1.05–1.29)	1.19 (1.13–1.26)
Uninsured	0.80 (0.67–0.96)	1.67 (1.47–1.91)	1.78 (1.54–2.04)	1.51 (1.39–1.63)
Race and Insurance type				
NHW with uninsured	Reference	Reference	Reference	Reference
NHW with non‐Medicaid	1.44 (1.19–1.75)	0.59 (0.51–0.68)	0.57 (0.49–0.66)	0.66 (0.61–0.72)
NHW with Medicaid	1.77 (1.37–2.27)	0.80 (0.68–0.93)	0.64 (0.54–0.76)	0.78 (0.71–0.85)
Asian with uninsured	1.61 (0.91–2.85)	1.03 (0.64–1.66)	1.20 (0.67–2.17)	0.99 (0.72–1.35)
Asian with non‐Medicaid	0.66 (0.53–0.84)	0.71 (0.59–0.85)	0.60 (0.49–0.75)	0.73 (0.63–0.83)
Asian with Medicaid	0.67 (0.47–0.94)	0.88 (0.68–1.13)	0.97 (0.67–1.41)	0.78–1.14)

## Appendix C

**Table:** Odds of lobectomy, mediastinal lymph node (MLN) evaluation, and cancer‐specific surgery and/or radiotherapy according to race and insurance type in each poverty group C

Variables	Lobectomy		MLN evaluation		Cancer‐directed surgery/RT	
	OR_adj_	95% CI	OR_adj_	95% CI	OR_adj_	95% CI
Percent of county below poverty <10						
Race						
NHW	1.00	Reference	1.00	Reference	1.00	Reference
Asian	1.75	1.21–2.52	0.99	0.82–1.20	0.88	0.69–1.12
Insurance type						
Uninsured	1.00	Reference	1.00	Reference	1.00	Reference
Non‐Medicaid	0.79	0.43–1.46	1.87	1.39–2.51	1.60	1.13–2.26
Medicaid	0.73	0.36–1.46	1.15	0.81–1.61	0.98	0.66–1.45
Race & Insurance type						
NHW & Uninsured	1.00	Reference	1.00	Reference	1.00	Reference
NHW & Non‐Medicaid	0.84	0.45–1.56	1.87	1.37–2.55	1.48	1.02–2.14
NHW & Medicaid	0.84	0.41–1.71	1.24	0.86–1.78	0.88	0.57–1.34
Asian & Uninsured	NA	NA	1.11	0.40–3.08	0.44	0.16–1.22
Asian & Non‐Medicaid	1.52	0.74–3.14	1.94	1.35–2.79	1.30	0.83–2.04
Asian & Medicaid	0.88	0.29–2.63	0.82	0.47–1.42	0.93	0.50–1.73
Percent of county below poverty 10–12						
Race						
NHW	1.00	Reference	1.00	Reference	1.00	Reference
Asian	1.37	0.99–1.91	0.84	0.70–0.99	0.67	0.55–0.81
Insurance type						
Uninsured	1.00	Reference	1.00	Reference	1.00	Reference
Non‐Medicaid	0.78	0.47–1.31	1.92	1.53–2.41	2.02	1.59–2.56
Medicaid	0.62	0.35–1.08	1.01	0.78–1.30	1.28	0.98–1.66
Race & Insurance type						
NHW & Uninsured	1.00	Reference	1.00	Reference	1.00	Reference
NHW & Non‐Medicaid	0.97	0.78–1.20	1.83	1.45–2.31	1.91	1.49–2.46
NHW & Medicaid	0.62	0.35–1.08	0.96	0.74–1.25	1.16	0.88–1.54
Asian & Uninsured	NA	NA	0.41	0.16–1.06	0.35	0.16–0.76
Asian & Non‐Medicaid	0.98	0.53–1.81	1.59	1.18–2.13	1.24	0.90–1.72
Asian & Medicaid	1.39	0.48–4.05	0.79	0.52–1.21	1.02	0.65–1.61
Percent of county below poverty 13–16.49						
Race						
NHW	1.00	Reference	1.00	Reference	1.00	Reference
Asian	2.65	1.51–4.64	1.16	0.92–1.46	0.84	0.64–1.12
Insurance type						
Uninsured	1.00	Reference	1.00	Reference	1.00	Reference
Non‐Medicaid	1.13	0.65–1.98	2.19	1.70–2.82	1.39	1.06–1.82
Medicaid	0.58	0.32–1.06	1.23	0.93–1.63	0.94	0.70–1.27
Race & Insurance type						
NHW & Uninsured	1.00	Reference	1.00	Reference	1.00	Reference
NHW & Non‐Medicaid	1.13	0.65–1.97	2.03	1.57–2.62	1.31	0.99–1.73
NHW & Medicaid	0.60	0.32–1.10	1.18	0.89–1.58	0.92	0.68–1.25
Asian & Uninsured	NA	NA	0.13	0.02–1.02	0.35	0.13–0.97
Asian & Non‐Medicaid	3.45	1.49–8.00	2.68	1.88–3.82	1.31	0.86–1.99
Asian & Medicaid	0.91	0.27–3.06	0.99	0.53–1.87	0.54	0.28–1.03
Percent of county below poverty ≥16.5						
Race						
NHW	1.00	Reference	1.00	Reference	1.00	Reference
Asian	1.33	1.01–1.77	1.03	0.89–1.20	0.82	0.69–0.96
Insurance type						
Uninsured	1.00	Reference	1.00	Reference	1.00	Reference
Non‐Medicaid	1.05	0.78–1.40	1.92	1.67–2.21	1.76	1.53–2.02
Medicaid	0.85	0.62–1.16	1.10	0.94–1.28	1.06	0.92–1.24
Race & Insurance type						
NHW & Uninsured	1.00	Reference	1.00	Reference	1.00	Reference
NHW & Non‐Medicaid	1.06	0.79–1.43	1.90	1.64–2.19	1.72	1.49–1.99
NHW & Medicaid	0.90	0.65–1.24	1.10	0.94–1.29	1.06	0.91–1.24
Asian & Uninsured	1.97	0.57–6.81	0.91	0.53–1.55	0.65	0.39–1.09
Asian & Non‐Medicaid	1.58	1.02–2.47	2.10	1.69–2.62	1.54	1.20–1.97
Asian & Medicaid	0.73	0.39–1.37	0.95	0.68–1.32	0.77	0.56–1.97

NHW, non‐Hispanic White.

^a^ Stage IV patients were excluded from data analysis, and multivariable model was adjusted for age, marital status, rural–urban area, percent of county below poverty, pathology results, and stage of disease, and analyzed under stratification of identical SEER registry by multilevel logistic regression model.

## Appendix D

**Table:** Hazard ratios of overall mortality according to race, insurance type and interaction between race and insurance type in each poverty groups of patients, analyzed by multilevel parametric survival analysis adjusted by patient demographic, pathology result, stage of disease, and cancer‐specific treatment

Percent of county below poverty	<10	10–12.99	13–16.49	≥16.5
	HR_adj_(95%CI)	HR_adj_(95%CI)	HR_adj_(95%CI)	HR_adj_(95%CI)
Race				
Non‐Hispanic White	Reference	Reference	Reference	Reference
Asian	0.77 (0.70–0.84)	0.77 (0.72–0.83)	0.72 (0.65–0.79)	0.68 (0.64–0.72)
Insurance type				
Non‐Medicaid	Reference	Reference	Reference	Reference
Medicaid	1.30 (1.20–1.40)	1.37 (1.30–1.45)	1.31 (1.23–1.39)	1.30 (1.25–1.34)
Uninsured	1.19 (1.07–1.33)	1.38 (1.27–1.50)	1.16 (1.06–1.26)	1.30 (1.24–1.36)
Race and Insurance type				
NHW with uninsured	Reference	Reference	Reference	Reference
NHW with non‐Medicaid	0.86 (0.77–0.96)	0.73 (0.67–0.80)	0.86 (0.79–0.93)	0.77 (0.73–0.81)
NHW with Medicaid	1.08 (0.95–1.24)	1.01 (0.92–1.11)	1.15 (1.04–1.26)	0.99 (0.94–1.04)
Asian with uninsured	1.01 (0.71–1.42)	0.93 (0.67–1.28)	0.68 (0.48–0.96)	0.60 (0.48–0.75)
Asian with non‐Medicaid	0.64 (0.55–0.73)	0.57 (0.51–0.63)	0.67 (0.58–0.77)	0.51 (0.47–0.56)
Asian with Medicaid	0.96 (0.77–1.18)	0.76 (0.65–0.89)	0.66 (0.53–0.82)	0.73 (0.65–0.82)

## Appendix E

Adjusted parametric survival curves illustrating cancer‐specific survival by insurance status for Asian and non‐Hispanic White lung cancer patients living in the United States in each poverty group analyzed by mixed‐effects Weibull regression. *P* < 0.05.

## Appendix F

**Table:** Hazard ratios of overall mortality according to race, insurance type and interaction between race and insurance type in patients diagnosed with **adenocarcinoma**, analyzed by multilevel parametric survival analysis adjusted for patient demographic, pathology result, stage of disease, and cancer‐specific treatment

Overall mortality	HR_adj_	95% CI
Race		
Non‐Hispanic White	1.00	Reference
Asian	0.69	0.65–0.72
Insurance type		
Non‐Medicaid	1.00	Reference
Medicaid	1.36	1.31–1.42
Uninsured	1.28	1.21–1.36
Race and Insurance type		
Non‐Hispanic White with uninsured	1.00	Reference
Non‐Hispanic White with non‐Medicaid	0.77	0.73–0.82
Non‐Hispanic White with Medicaid	1.05	0.98–1.13
Asian with uninsured	0.59	0.48–0.73
Asian with non‐Medicaid	0.53	0.49–0.58
Asian with Medicaid	0.73	0.65–0.82

## Appendix G

**Table:** Distribution of insurance status according to race in each poverty group

Race	Asian			Non‐Hispanic White		
	Non‐Medicaid *N* (%)	Medicaid *N* (%)	Uninsured *N* (%)	Non‐Medicaid *N* (%)	Medicaid *N* (%)	Uninsured *N* (%)
Poverty						
<10	1305 (81.6)	225 (14.1)	70 (4.3)	5896 (81.6)	747 (10.3)	583 (8.1)
10–12.99	1297 (72.8)	401 (22.5)	84 (4.7)	14,579 (79.7)	2703 (14.8)	1004 (5.5)
13–16.49	658 (75.5)	154 (17.7)	59 (6.8)	8426 (74.2)	1955 (17.2)	971 (8.6)
≥16.5	1404 (64.9)	572 (26.4)	187 (8.7)	22,777 (68.8)	7334 (22.2)	2978 (9.0)
